# Evolution of bird sex chromosomes: a cytogenomic approach in Palaeognathae species

**DOI:** 10.1186/s12862-024-02230-5

**Published:** 2024-04-23

**Authors:** Príncia Grejo Setti, Geize Aparecida Deon, Rodrigo Zeni dos Santos, Caio Augusto Gomes Goes, Analía Del Valle Garnero, Ricardo José Gunski, Edivaldo Herculano Corrêa de Oliveira, Fábio Porto-Foresti, Thales Renato Ochotorena de Freitas, Fábio Augusto Oliveira Silva, Thomas Liehr, Ricardo Utsunomia, Rafael Kretschmer, Marcelo de Bello Cioffi

**Affiliations:** 1 Departamento de Genética e Evolução, Universidade Federal de São Carlos, 13565-905 São Carlos, SP Brazil; 2 Faculdade de Ciências, Universidade Estadual Paulista, 17033-360 Bauru, São Paulo, Brazil; 3 Campus São Gabriel, Universidade Federal do Pampa, 97307-020 São Gabriel, Rio Grande do Sul Brazil; 4 Laboratório de Citogenômica e Mutagênese Ambiental, Seção de Meio Ambiente, Instituto Evandro Chagas, 67030-000 Ananindeua, PA Brazil; 5 Instituto de Ciências Exatas e Naturais, Universidade Federal do Pará, 66075-110 Belém, PA Brazil; 6 Departamento de Genética, Universidade Federal do Rio Grande do Sul, 91501-970 Porto Alegre, RS Brazil; 7 Institute of Human Genetics, Jena University Hospital, Friedrich Schiller University, 07747 Jena, Germany; 8 Departamento de Ecologia, Zoologia e Genética, Instituto de Biologia, Universidade Federal de Pelotas, 96.010-610 Pelotas, RS Brazil

**Keywords:** Molecular cytogenetics, Evolution, Nascent sex chromosomes, satDNAs, Birds

## Abstract

**Background:**

Different patterns of sex chromosome differentiation are seen in Palaeognathae birds, a lineage that includes the ratites (Struthioniformes, Rheiformes, Apterygiformes, Casuariiformes, and the sister group Tinamiformes). While some Tinamiform species have well-differentiated W chromosomes, both Z and W of all the flightless ratites are still morphologically undifferentiated. Here, we conducted a comprehensive analysis of the ZW differentiation in birds using a combination of cytogenetic, genomic, and bioinformatic approaches. The whole set of satDNAs from the emu (*Dromaius novaehollandiae*) was described and characterized. Furthermore, we examined the in situ locations of these satDNAs alongside several microsatellite repeats and carried out Comparative Genomic Hybridizations in two related species: the greater rhea (*Rhea americana*) and the tataupa tinamou (*Crypturellus tataupa*).

**Results:**

From the 24 satDNA families identified (which represent the greatest diversity of satDNAs ever uncovered in any bird species), only three of them were found to accumulate on the emu’s sex chromosomes, with no discernible accumulation observed on the W chromosome. The W chromosomes of both the greater rhea and the emu did not exhibit a significant buildup of either C-positive heterochromatin or repetitive DNAs, indicating their large undifferentiation both at morphological and molecular levels. In contrast, the tataupa tinamou has a highly differentiated W chromosome that accumulates several DNA repeats.

**Conclusion:**

The findings provide new information on the architecture of the avian genome and an inside look at the starting points of sex chromosome differentiation in birds.

**Supplementary Information:**

The online version contains supplementary material available at 10.1186/s12862-024-02230-5.

## Introduction

The canonical model of sex chromosome evolution states that they are derived from an autosome pair when one of the homologs acquires a sex-determining gene, followed by the accumulation of sexually antagonistic mutations in this proto-sex chromosome [1–3]. Consequently, the recombination between the chromosomes is gradually reduced, followed by the accumulation of repetitive DNA sequences and heterochromatin in the differentiating W or Y chromosome [1, 4–7]. Repetitive DNAs are the first elements to accumulate in the ancient recombining early stages of the differentiation of the sex chromosomes, which can lead to a cytologically detectable heteromorphism between them (for a review, see [8, 9]. This accumulation usually involves transposable elements or satellite DNAs (reviewed in [10]). This process is evident even in sex chromosomes that are still evolving, such as those of *Drosophila miranda* [11], *Silene latifolia* [12, 13], or *Carica papaya* [14]. Therefore, repeat accumulation may represent an early stage in modifying the sex-specific chromosome, even before the genes start to degenerate [15]. Nowadays, cytogenetics combined with other up-to-date genomic methods like high-throughput sequencing provides a more detailed overview of the sex chromosomes’ evolutionary path [16–18].

**Fig. 1:**
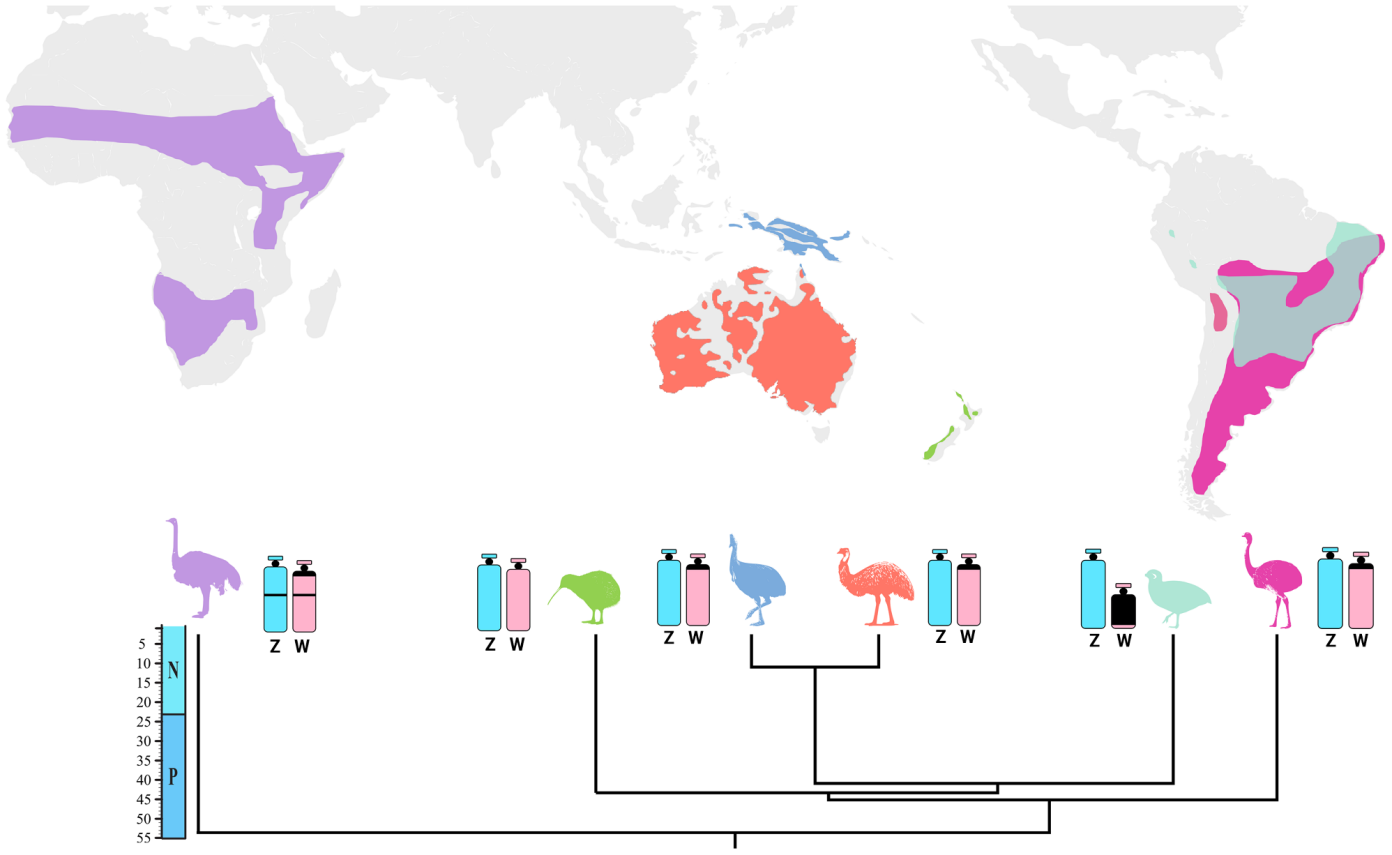
Geographic distribution of Palaeognathae, showing color-coded species based on their geographical occurrence: *Struthio* (purple), *Apteryx* (green), *Casuarius* (blue), *Dromaius* (orange), *Crypturellus* (light green), and *Rhea* (pink). The dated species tree was obtained from [20]. Representative idiograms for the Z (light blue) and W (light red) sex chromosomes are provided, together with information on their C-banding (black) patterns (except for *Apteryx*). Data was retrieved from [25–27] and present data). A geological scale with key periods is depicted on the left (N = Neogene; P = Paleogene)

With more than 11.000 species, birds represent a very diverse group of tetrapod vertebrates [19–21]. They are divided into two clades, Palaeognathae and Neognathae, based on palatal anatomy. Palaeognathae birds encompass the ‘flightless’ ratites, including the kiwis (Apterygiformes), emus and cassowaries (Casuariiformes), rheas (Rheiformes), and ostriches (Struthioniformes), and also the ‘flying’ tinamous (Tinamiformes) [20] While Palaeognathae is considered a monophyletic group [20], new pieces of evidence (i.e.: molecular data, including convergent regulatory evolution, biogeographic and phylogenomic relationships of extinct paleognaths) point that the flightless Palaeognathae (former Ratites) are paraphyletic, since there were many parallel losses of flight throughout the Palaeognathae lineage [22–24]. The current geographical distribution of Palaeognathae is shown in (Fig. 1).

Looking at the Palaeognathae group from a cytogenetic perspective, it is noteworthy that some Tinamiform species exhibit significant variability in the differentiation of the W chromosome, while the ratites still present large undifferentiated ones harboring a poor heterochromatin accumulation [28–31] (Fig. 1). For example, despite being over 100 million years old, the W chromosome of the ostrich (*Struthio camelus*) is still 65% the size of the Z chromosome [32]. Paleognaths have significant nondegenerate sections (also known as pseudoautosomal regions, or PARs) on their sex chromosomes, in contrast to other birds [32, 33]. In ostrich, just one-third of the Z chromosomes do not recombine with the W, while in the emu, the non-recombining region is even shorter, being confined to the W centromere and the Z short arms, which contain the DMRT1 gene [30, 34, 35].

The emu (*Dromaius novaehollandiae*) is one of the most iconic ratite species, being the first bird species to have had its chromosomes hybridized with chicken macrochromosomes paints [36]. Emu, rheas, and other ratites have been deeply explored to understand the evolutionary path of birds due to their basal position in avian phylogeny and ancestrally similar karyotypes. The findings include (i) the arrangement and organization of chromosomes in the nucleus [27, 30, 34]; (ii) the low number of BAC-scale chromosomal rearrangements and deletions [37]; and (iii) the changes in chromatin conformation in the ancient recombining sex chromosomes differentiation [30].

Here, we conducted a comprehensive analysis of the ancient recombining ZW chromosomes among birds, using the emu - *D. novaehollandiae* - as a model. We aimed to answer the following questions: (i) are repetitive DNAs largely accumulated in the sex chromosomes, given that they are still large undifferentiated? (ii) does the W chromosome accumulate unique repetitive sequences not found on the Z? (iii) are these same repeats accumulated in the Z and/or W chromosomes of the emu also conserved in the W chromosomes of other closely related species? In that regard, we compared the intragenomic differences between males and females. We used cytogenetic and genomic approaches to analyze their satellitome composition and the putative involvement of these satellite DNAs and microsatellites in the initial stages of the W chromosome differentiation. In addition, these sequences were also hybridized in the chromosomes of two other Palaeognathae species: the greater rhea (*Rhea americana*, Rheiformes, Rheidae), which also exhibits morphologically undifferentiated ZW chromosomes, and the tataupa tinamou (*Crypturellus tataupa*, Tinamiformes, Tinamidae), a species included in a sister group of the ratites with a well-differentiated W chromosome.

## Results

### Karyotype and C-banding

First, we investigated and confirmed that the 2n for all three Palaeognathae species investigated was 80, which corroborated earlier information for these species [25, 29, 38]. For all species, the same pattern for the autosomes was observed, i.e.: while several microchromosomes are heterochromatic, the majority of macrochromosomes exhibit C-positive heterochromatin at centromeric regions. Nevertheless, the tataupa tinamou’s W chromosome was fully heterochromatic, in contrast to the Z chromosomes of all species and the W chromosomes of the emu and the greater rhea, which showed very weak C-positive heterochromatic blocks in the centromeric region (Supplementary Fig. 1).

### Satellite DNA content of the emu

To start the answers proposed in this work, we identified 24 satDNA families in the emu genome, designated as DnoSatDNAs and numbered from the most to the least abundant in the genome (Supplementary Table 1). The Repeat unit length (RUL) had a median of 214 bp and a range of 31 to 5.881 bp. Long satellites (> 100 bp) were the most common type, comprising 20 satDNAs. The A + T percentage was 41,375%, indicating a predominance of the G + C base pairs. Supplementary Fig. 2 displays the repeat landscapes illustrating the distribution and divergence of all the DnoSatDNA families.

Although, in general, most satDNA sequences have a notable A + T content [39, 40], this does not occur in the emu, as has also been previously found in birds [41]. We found that 23 of the 24 DnoSatDNAs contain more than 50% of G + C base pairs, which does not occur in some bird genomes [41], indicating a possibly specific trait. The only exception was the DnoSat02, which is AT-rich (56.60%) and represents the largest satDNA (5881 RUL).

### Autosomal distribution of repetitive DNAs in the emu

**Fig. 2:**
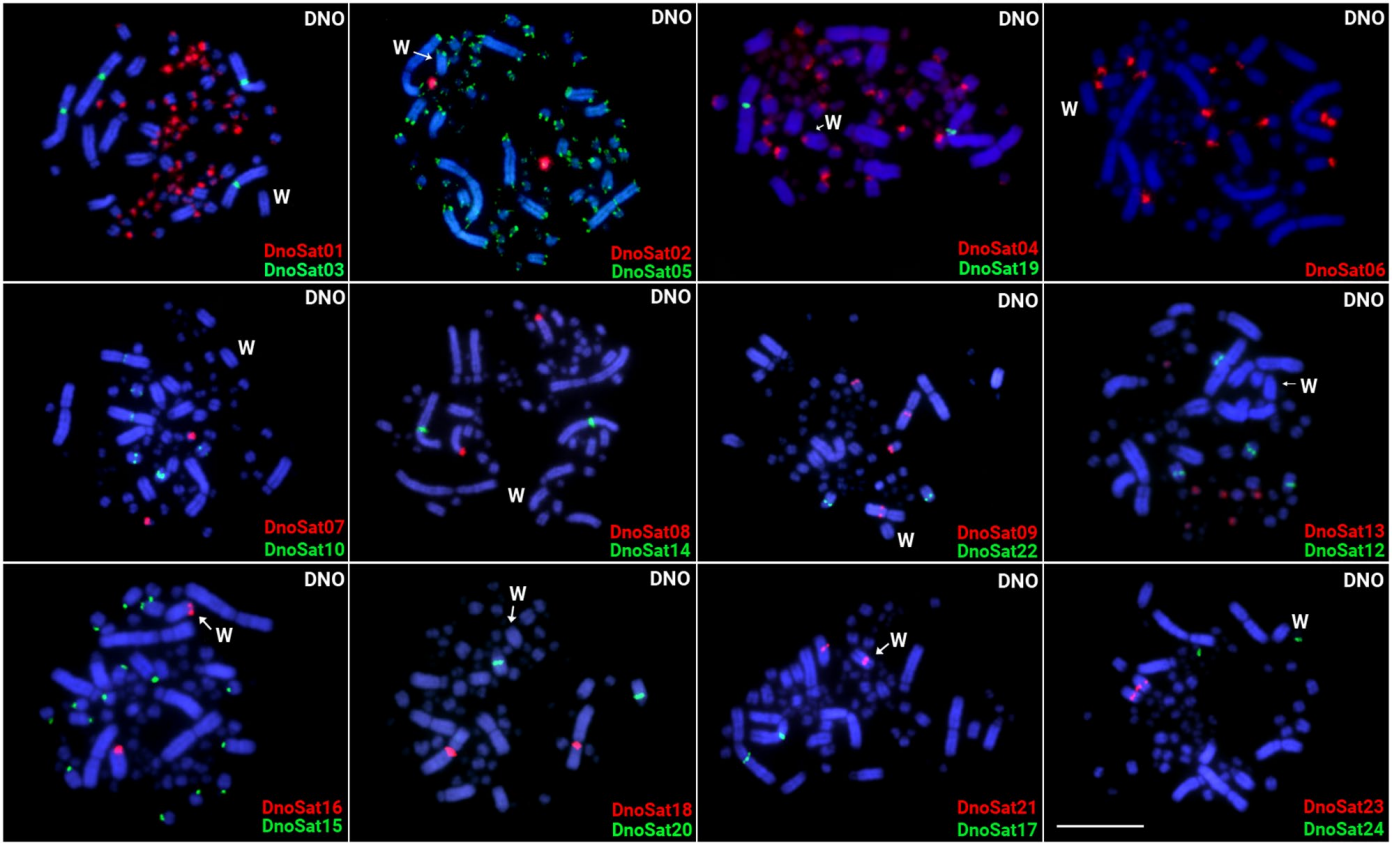
Female metaphase plates of the emu highlighting the chromosomal location of 23 DnoSatDNAs. Their family names are indicated in the lower right corner, in green (Atto488-dUTP labeled) or red (Atto550-dUTP labeled). While the W chromosome was appropriately identified by a sequential hybridization with the microsatellite (GA)n, the Z chromosome could not be properly identified based on its morphology. Bar = 10 μm

**Fig. 3:**
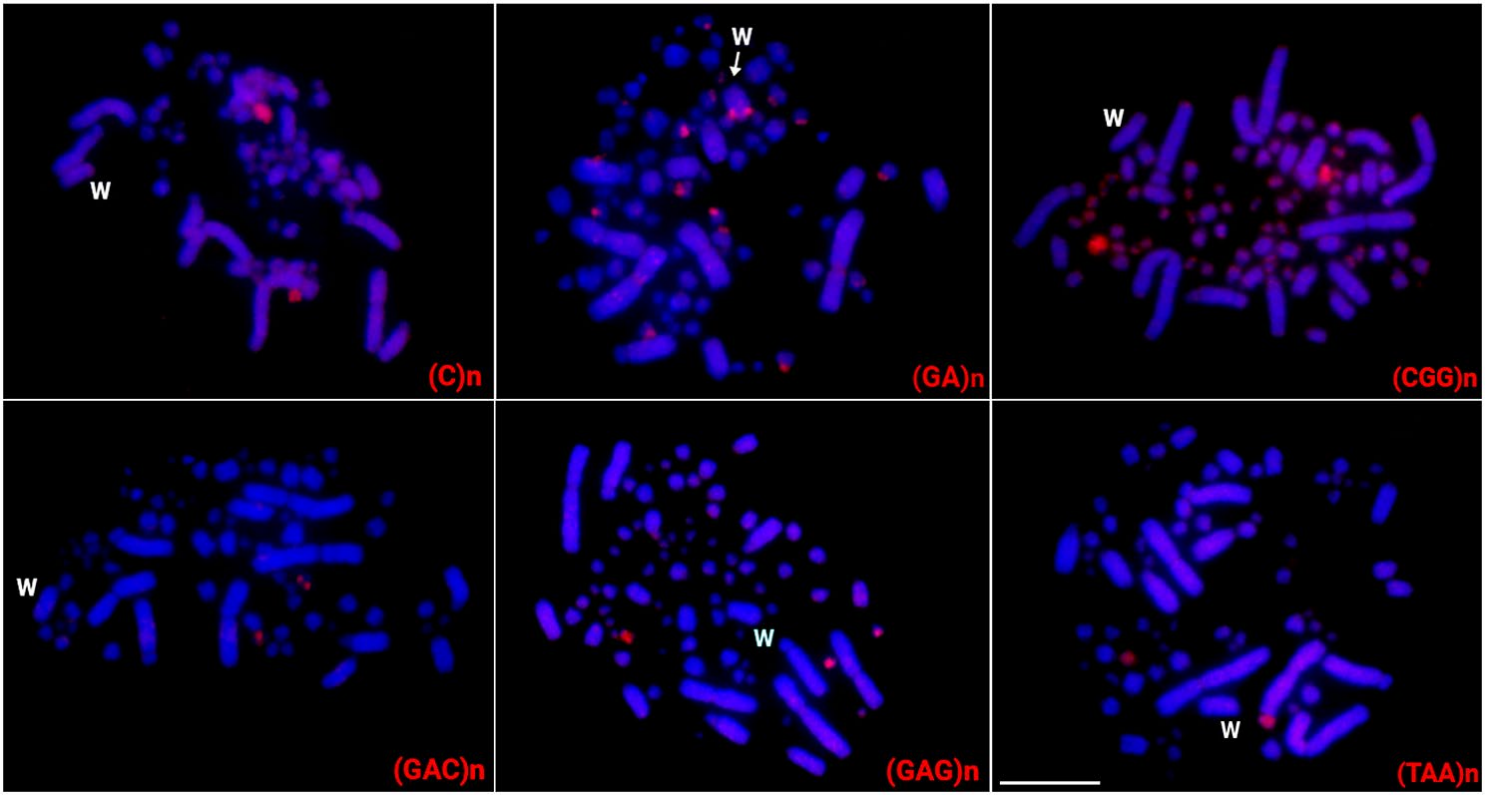
Female metaphase plates of the emu highlighting the chromosomal location of microsatellite repeats, indicated in the lower right corner in red. While the W chromosome was appropriately identified by a sequential hybridization with the microsatellite (GA)n, the Z chromosome could not be properly identified based on its morphology. Bar = 10 μm

The in situ investigations highlighted that the majority of DnoSatDNAs (20 out of 23), were exclusively located on autosomes. The DnoSat02, 07, and 24 hybridized only in a pair of microchromosomes, while DnoSat8, 14, 17, 18, 20, 22, and 23 hybridized in a pair of macrochromosomes. DnoSat05 deviated from this standard and hybridized in the telomeric region of all chromosomes. In the other hand, DnoSat01, 03, 06, 09, 10, 12, 13, 15, and 19, were mapped in multiple micro- or macrochromosomes (Fig. 2). On the other hand, only 06 out of 17 microsatellites tested displayed positive FISH signal in the emu chromosomes. Except for (GA)n, which displays signals in many autosomes and in the W chromosome (Please see Supplementary Fig. 3), all the others were exclusively mapped to autosomes, mostly in microchromosomes (Fig. 3).

### Distribution of repetitive sequences in the Emu sex chromosomes

Only three DnoSatDNAs, named DnoSat04, DnoSat16 and DnoSat21, and the microsatellite (GA)n showed hybridization in the emu sex chromosomes. Despite also being accumulated in many autosomes, the presence of a conspicuous (GA)n signal in the pericentromeric region of a single macrochromosome, unique to females (Supplementary Fig. 3), allows us to identify it as the W chromosome. DnoSat16 and DnoSat21 were mapped in both Z and W chromosomes. Although the Z chromosome cannot be properly identified based on its morphology, given that both DnoSatDNAs only show two hybridization signals, one of which is located in the W chromosome, we may infer that the other signal is located on the Z chromosome. On the other hand, DnoSat04 displays signals in the W chromosome and several autosomes, in both micro- and macrochromosomes. Therefore, in this case, its presence on the Z chromosome cannot be confirmed (Figs. 2 and 3).

### Repetitive sequences in the other Palaeognathae species

None of the DnoSatDNAs produced FISH signals on the chromosomes of the greater rhea and tatuapa tinamou, showing that these same repeats are not conserved in the W chromosomes of these two closely related species.

**Fig. 4:**
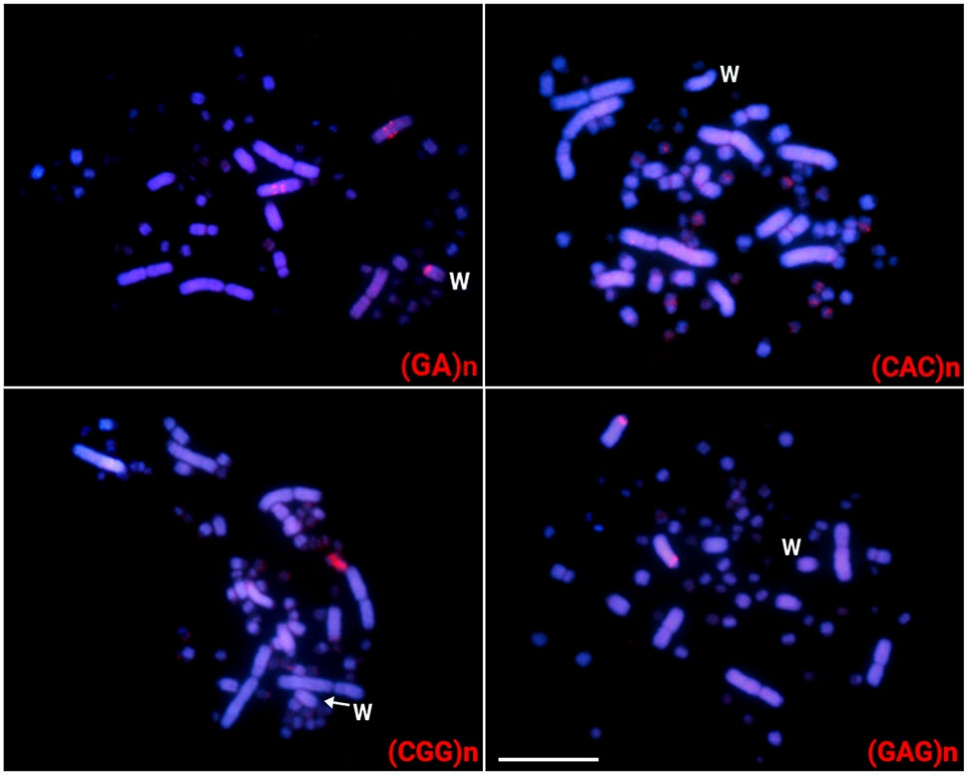
Female metaphases of the greater rhea highlighting the chromosomal location of microsatellite repeats. The microsatellites are indicated in the lower right corner in red. While the W chromosome was appropriately identified by a sequential hybridization with the microsatellite (GA)n, the Z chromosome could not be properly identified based on its morphology. Bar = 10 μm

Out of the 17 microsatellites tested, only four [named (CGG)n, (CAC)n, (GA)n and (GAG)n, ] display positive FISH signals in the greater rhea chromosomes. The (GA)n microsatellite was mapped in the W chromosome and in the third biggest macrochromosome pair. (GAG)n was found in a macrochromosome autosomal pair, while (CAC)n and (CGG)n showed positive signals only in microchromosomes (Fig. 4).

**Fig. 5:**
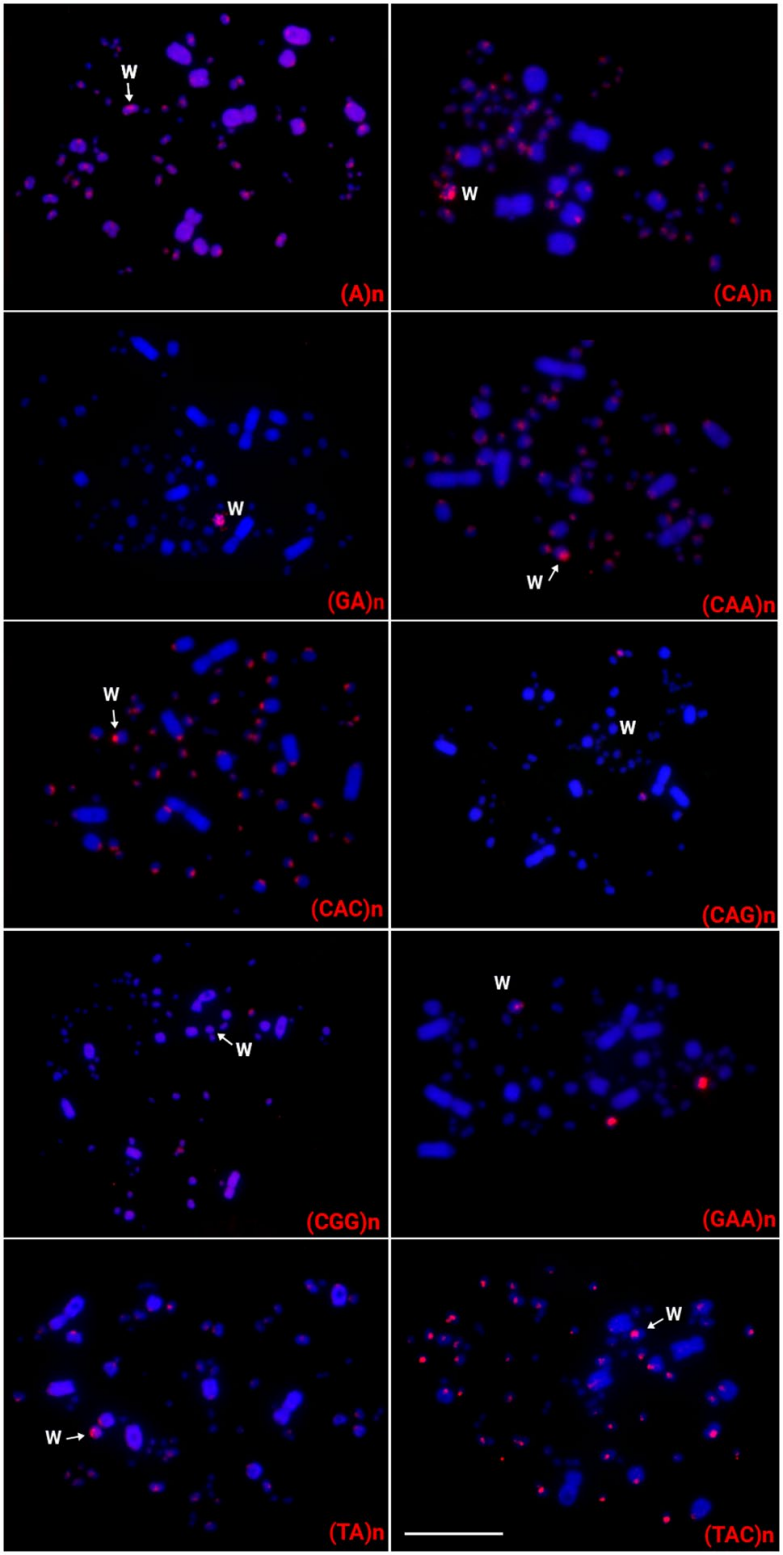
Female metaphases of the tataupa tinamou highlighting the chromosomal location of microsatellite repeats. The microsatellites are indicated in the lower right corner in red. While the W chromosome was appropriately identified by a sequential hybridization with the microsatellite (GA)n, the Z chromosome could not be properly identified based on its morphology. Bar = 10 μm

Differently from the scenario found for both ratite species (emu and greater rhea), nine microsatellite sequences were highly accumulated in the tataupa tinamou chromosomes. Except for the (CGG)n and (CAG)n microsatellites, which were mapped in just one pair of microchromosomes, all the other ones were mapped in the W chromosome. While the microsatellite (GA)_15_ was exclusively mapped in the W chromosome, (A)n, (CA)n, (CAA)n, (CAC)n, (GAA)n, (TA)n, and (TAC)n were also present in other macro and microchromosomes (Fig. 5).

### Amplification of DnoSatDNA in *Rhea americana*

Among all the 24 DnoSatDNAs investigated, the DnoSat01, 03, 08, 14, 16, 20, and 21 satellites were also present in the greater rhea genome. However, after FISH studies, none of them produced hybridization signals in its chromosomes (data not shown).

### Comparative genomic hybridization

**Fig. 6:**
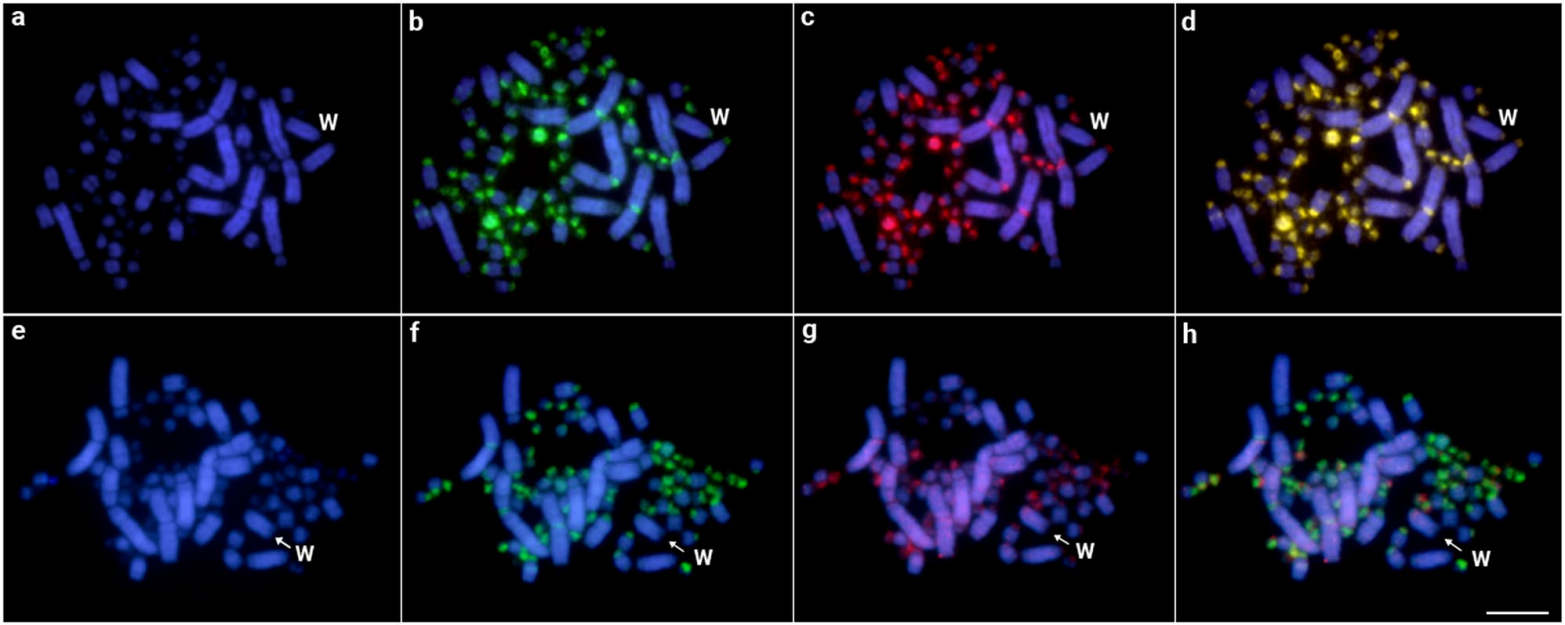
Intraspecific genomic hybridization with emu male and female gDNA probes hybridized in female metaphase chromosomes (**a-d**). (**a**) DAPI-stained metaphases of the emu female, (**b**) hybridization pattern of the male-derived probe (green), (**c**) hybridization pattern of the female-derived probe (red), and (**d**) merged images of both genomic probes and DAPI staining. Interspecific genomic hybridization between the emu and the greater rhea (**e-h**). (**e**) DAPI-stained metaphases of the emu female, (**f**) hybridization pattern of the emu female-derived probe (green), (**g**) hybridization pattern of the greater rhea female-derived probe (red), and (**h**) merged images of both genomic probes and DAPI staining. While the W chromosome was appropriately identified by a sequential hybridization with the microsatellite (GA)n, the Z chromosome could not be properly identified based on its morphology. Bar = 5 μm

Finally, when analyzing specific sequences for each sex, we identified multiple overlapping regions, mostly in the centromeric regions of all macro- and microchromosomes of the emu. It did not reveal any specific female sequence in the W chromosome. Interspecific comparisons between the emu and the greater rhea females evidenced the accumulation of emu-specific sequences in most chromosomes and some microchromosomes (Fig. 6).

## Discussion

### General organization of SatDNAs in the emu genome

Birds frequently have shorter genomes because of the decrease in their repetitive DNA content and in the number of genes related to metabolic needs and flight, which exposes these species to strong stabilizing selection [42–45]. Consequently, it is presumed that most bird species have a small number of repetitive sequences, leading to a smaller number of satDNA families [46]. However, this hypothesis is supported by limited available data [41, 47, 48]. This scenario completely changes regarding some birds unable to fly. Besides having denser and heavier bones, flightless birds present larger genomes with a higher amount of repetitive DNAs [49–51]. Accordingly, we found 24 satDNA sequences in the emu (which corresponded to ∼6% of their genome), a higher number than those previously documented, which are predominantly long, (i.e., exceeding 100 bp), with a nearly equal female-to-male abundance ratio (Table 1). To obtain a more precise outcome, we investigated the DnoSat02 occurrence in some other species using the assembled genomes from the emu (GCF_003342905.1),: *Apteryx rowi* (GCF_003343035.1), *Gavialis gangeticus* (GCF_001723915.1), and *Gallus gallus* (GCF_016699485.2). This sequence was only found in the emu, with no tandem repeats in any of the other species.

Except for DnoSat04, DnoSat16 and DnoSat21, all the other DnoSatDNAs were exclusively mapped to autosomes, in both macro- and microchromosomes, but preferentially in the last ones (Fig. 2). Comparable results were also discovered in the lesser rhea (*Pterocnemia pennata*) and in the great rhea (*Rhea americana*) utilizing genomic DNA digestion with a restriction endonuclease [52]. This condition is also linked to the reduction of the avian genome when compared to that of reptiles, due to the considerable correlation between GC content and the greater tendency to remove non-coding regions [30]. On the other hand, although seven DnoSatDNAs were also present in the great rhea genome (data not shown), none of them displayed positive hybridization signals after FISH investigations. As previously demonstrated in insects and plants [53–55], related species may have an ancestral collection of different conserved satDNA families that are differently amplified in each lineage. As a result, only a few copies of these sequences are found among related species [17]. The evolution of satDNAs can be explained by two complementary hypotheses: (i) the library one, which addresses the independent expansion, contraction, and homogenization of satDNAs in divergent species; (ii) the concerted hypothesis, which considers recombination processes leading to duplication, exclusion, and homogenization of common satDNAs in divergent species [17, 56–59]. Together, these hypotheses can explain how satDNAs evolved and why their long-term conservation is, in fact, not expected.

### Ancient recombining ZW sex chromosomes evolution in birds

Even though the sex chromosomes have been extensively investigated since the early 1900s [4], there are still a lot of unanswered questions, and new studies are consistently identifying new pathways for their evolution [60–64]. Among birds, most species contain a well-differentiated ZZ/ZW sex chromosome system, with small and heterochromatic W chromosomes (reviewed in [46, 65, 66]). In contrast, ratite birds present morphologically undifferentiated sex chromosomes, with both Z and W being still morphologically similar [27, 67–69]. One of the best ways to analyze the steps of sex chromosome differentiation is to investigate them in groups where this process is still developing or in the nascent stage as in ratite species.

In the present study, the combined cytogenetic and genomic approaches demonstrated that the W chromosomes of the emu and in the great rhea are large undifferentiated. In addition to being morphologically like the Z and poor in the heterochromatin content, there is no specific accumulation of repeats in the W chromosomes. In the emu, only three DnoSatDNAs were found in the sex chromosomes, with no discernible difference in accumulation on the W chromosome (Fig. 2). The (GA)n microsatellite is the sole exception, showing a distinct accumulation pattern on the W chromosome in both species (Figs. 3 and 4). Besides, no substantial molecular differentiation was obtained in their W chromosomes after intra- and interspecific CGH experiments (Fig. 6).

The primary cause of the differentiation of the Y or W chromosomes is the cessation of recombination between a significant portion, if not all, of a previously undifferentiated X/Y or Z/W sex pair [70–72]. Likewise, heterochromatinization is closely related to the accumulation of repetitive DNA sequences on the W or Y chromosomes, contributing to their morphological differentiation and the origin of a short PAR [73]. Chromosome painting with a chicken Z-derived probe produced FISH signals in the entire length of the emu Z chromosome, showing their large homology. These paints also produced signals along most of the W chromosome, except for a small region on its short arm and the centromeric region, demonstrating the large homology shared by the emu sex pair [36]. In this scenario, just two DnoSatDNAs (16 and 21) were exclusively shared by both Z and W chromosomes, and probably located in the PAR region. Despite their similar distribution, *in silico* analysis suggests that the DnoSat18 is twice as abundant in females, whereas the female-male ratio of the DnoSat21 is 1.08 (Supplementary Table 1), indicating a possible accumulation of the DnoSat18 and an early differentiation of the emu W chromosome.

On the other hand, despite being phylogenetically related and belonging to the same bird group (Palaeognathae), the tataupa tinamou displays a well-differentiated and heterochromatic-rich W chromosome that accumulates large amounts of microsatellite repeats, although not preserving any DnoSatDNAs (Fig. 5). Previous data from lizards, plants, and fishes emphasize that microsatellites represent the very early colonizers of new Y/W sex chromosomes after the recombination of the sex pair is stopped [74–76]. In turn, contrasting to other Tinamiformes species, the tinamou tatuapa exhibits a distinctive feature in the W chromosome, which is completely heterochromatic, as demonstrated by [77] and [25], as well as by our current investigation. Because of this heterochromatic nature, the recombinant region between the sex chromosomes of the tatuapa tinamou is notably smaller compared to other species. This region is confined to the terminal segment of the long arm of the W chromosome, in contrast to the elegant crested tinamou (*Eudromia elegans*), where the recombinant region encompasses one-fourth of the W chromosome length [25]. Although the emu and the tataupa tinamou belong to the Palaeognathae group, they diverged at approximately 62 Mya [78], which helps to understand their contrasting mode of sex chromosome evolution.

Very ancient and morphologically undifferentiated sex chromosomes have also been documented, such as the ones present in the sturgeons (∼180Mya) and osteoglossiforms (∼200 Mya) fishes [79, 80], as well as in the ratites (> 130 Mya) [81]. Ratites, however, stand out among the aforementioned cases because, in contrast to the other examples, the great majority of bird species have established a well-differentiated ZZ/ZW sex system. Furthermore, the possibility of these chromosomes undergoing a turnover event over time is quite low [82]. So, what sustains their long-term large undifferentiation? The prevalence of homomorphic sex chromosomes for more than 130 Mya among ratite species and the evolutionary forces that may have hindered their W chromosome differentiation can be explained by two hypotheses: (i) the first one relates how the sexual bias affects the gene expression of the Z chromosome, both in its PAR and non-recombinant regions. This bias can limit the offspring of a sexually antagonistic allele to the sex that benefits, leading to an evolutionary process that produces species with homomorphic chromosomes [83, 84]; (ii) The second hypothesis postulates that the recombination near the PAR region can break the association between the sex-linked and the PAR regions which, in turn, minimizes the influence of sexual linkage with nearby genes and reduces the probability of sex-specific mutations [32]. With this particular event, a high recombination rate between sex chromosomes likely causes the large PAR extent, as shown in ostrich [32].

## Conclusion

In this study, we characterized the complete satDNA library, commonly referred to as satellitome, of the emu and conducted a comparative analysis with two other Palaeognathae species: the great rhea and the tataupa tinamou. We showed the occurrence of 24 distinct satDNA sequences, a notably higher number compared to previously documented cases in other avian species. However, no large accumulation of C-positive heterochromatin and repetitive DNAs was observed in the W chromosomes of both the emu and the greater rhea, highlighting that they have escaped from a large differentiation at the molecular level. The tataupa tinamou, on the other hand, presents a contrasting scenario given its highly differentiated W chromosome, which accumulates several DNA repeats. The results allow us to have an inside look at the very early stages of sex chromosome differentiation in birds, in addition to offering fresh insights into the architecture of the avian genome.

### Methods

#### Sampling, chromosomal preparation, and C-banding

Individuals of *Dromaius novaehollandiae* (DNO; Emu); *Crypturellus tataupa* (CTA; Tataupa tinamou) and *Rhea americana* (RAM; greater rhea) were analyzed in this study (Table 1). All these specimens were collected under the permission of the Brazilian environmental agency ICMBio/SISBIO (61047-2 and 68443-2) and SISGEN (A96FF09). The *Dromaius novaehollandiae* and *Rhea americana* specimens were obtained from ex-situ individuals, while the *Crypturellus tataupa* ones were sampled in their natural habitats. Fibroblast cell cultures were used to acquire chromosomes from the feather pulp, according to [85]. In general, cells were grown in flasks (25 cm^2^) containing DMEM culture media (GIBCO), fetal bovine serum (15% GIBCO), and 1% penicillin (10,000 units/mL)/streptomycin (10,000 g/mL) (GIBCO). The C-positive heterochromatin was detected following [86] and the slides were further counterstained with propidium iodide (200 ng/ml in 2x SSC, Sigma). All experiments followed the guidelines and were approved by the Ethics Committee on Animal Experimentation of the Universidade Federal do Pampa, Brazil (Process number CEUA 018/2014).

**Table 1:** Species, locality, number, and sex of individuals (N) used in the present study

Species	Location	N
*Dromaius novaehollandiae* (DNO; Emu)	Glorinha (RS)	(01♀; 01♂)
*Crypturellus tataupa* (CTA; Tataupa tinamou)	Porto Vera Cruz (RS)	(01♀; 02♂)
*Rhea americana* (RAM; greater rhea)	Sapucaia do Sul (RS)	(01♀; 0♂)

RS: Rio Grande do Sul, Brazilian State.

### DNA extraction and genome sequencing

The gDNA from the male and female emu specimens were extracted following the protocol by [87]. Both gDNA samples were sequenced using the BGISEQ-500 platform (paired-end 2 × 150 bp), with a 3x coverage normally required for satellite assembly [16, 88]. Raw reads were deposited on the SRA-NCBI and are available under the accession numbers: SRR26815296-SRR26815299.

### Bioinformatic analyses: the characterization of emu satellitome

We applied the satMiner [16] bioinformatic pipeline to describe the satellitome of *D. novaehollandiae*. After quality and adapter trimming using Trimmomatic [89], we performed a random selection of 2 × 500,000 reads to characterize the satellitome using the TAREAN tool [90]. Then, putative satDNA sequences found by TAREAN were filtered from the genomic libraries using the software Deconseq [91], and a new subsample of 2 × 500,000 reads were randomly selected, repeating the process. We repeated these iterations until no satDNAs were found. Other repetitive elements, such as multigene families, were removed from the putative satDNAs described by TAREAN, and a homology search was performed using RepeatMasker software [92] to remove possible redundancies (sequences with > 95% similarity were considered the same variants) and to group other sequences in variants of the same satDNA (similarity between 80% and 95%), or superfamilies (similarity between 50% and 80%), as suggested in [16]. The sequences were deposited on the GenBank with the accession numbers OR813804-OR813827.

### Estimating the abundance and diversity of SatDNAs

The abundance of each satDNA was calculated with RepeatMasker [92] using the “cross-match” option. For that, we used 2 × 5,000,000 reads and mapped that were mapped against the satDNA catalogue. Their genetic distances were calculated using the script calcDivergenceFromAlign.py. We used the Kimura-2 parameter to build repeat landscapes to illustrate the distances between each satDNA family.

### Primer design and DNA amplification by polymerase chain reaction

We designed primers for 21 out of the 24 DnoSatDNAs that were characterized. A total of 34 cycles (with initial denaturation at 95 °C for 45 s, initial annealing at 58–64 °C for one-minute, initial extension at 72 °C for one minute, and final extension at 72 °C for 7 min) were performed using 10 ng of DNA for each satellite. Positive DNA amplification was confirmed by agarose gel electrophoresis and quantification using the ThermoFisher NanoDrop spectrophotometer (ThermoFisher Scientific). To verify the presence of DnoSatDNA in the greater rhea, PCR experiments were performed using its gDNA as a template, following the same conditions described above for the emu.

### Fluorescence in situ hybridization (FISH)

After amplification, each satDNA was labeled with the Atto550-dUTP (red) or Atto488-dUTP (green) fluorophores using a Nick-Translation Kit (Jena Bioscience, Jena, Germany). Satellite DNAs with repeat unit lengths smaller than 40 bp (DnoSat04, DnoSat06, and DnoSat19) were directly labeled with Cy3 at the 5’ end during the synthesis by ThermoFisher (ThermoFisher Scientific). In addition, 17 other microsatellites, [(C)n, (A)n, (GA)n, (CA)n, (GC)n, (TA)n (CAA)n, (CAG)n, (CAT)n, (GAG)n, (TAA)n, (TAC)n, (GAC)n, (CGG)n, (CAC)n and (GAA)n, (GATA)n], labeled with Cy3 during synthesis (VBC Biotech, Vienna, Austria), were also used as probes. All probes were hybridized in the metaphase chromosomes of the emu, tataupa tinamou, and greater rhea following the protocol described by [93].

### Comparative genomic hybridization: experimental design and probe preparation

The genomic DNAs from male and female emu specimens and the greater rhea female were extracted from feather pulp tissues by the standard phenol-chloroform method [87]. Two experimental designs were used for this study. The first set of experiments was designed to examine the extent of genetic differentiation on the W chromosome intra-specifically, in the emu. The gDNAs of male and female specimens were labeled in red and green, respectively, with Atto550-dUTP and Atto488-dUTP, using nick-translation (Jena Biosciences), and were hybridized against the female chromosome complement. To block the shared repetitive sequences, we used unlabeled C0t-1 DNA (i.e., gDNA fraction enriched in highly and moderately repetitive sequences), prepared according to [94]. The ratio of the probe vs. C0t-1 DNA was chosen based on previous investigations of our research group [95–100]. The final hybridization mixture for each slide was composed of both male and female genomic probes (500 ng each) supplemented with 3 µg of male-derived C0t-1 DNA.

The second set of experiments was designed to compare the molecular composition of the W chromosome of the emu and the greater rhea. To achieve this, the female gDNAs of both species were labeled in red (the emu) and green (greater rhea) with Atto550-dUTP and Atto488-dUTP, using nick-translation (Jena Biosciences), and were hybridized against the female chromosome of the emu. We used unlabeled C0t-1 DNA of both species to block the shared repetitive sequences, applying a probe ratio vs. C0t-1 DNA based on our previous investigations, as related above. The final hybridization mixture for each slide was composed of female genomic probes (500 ng of each species) supplemented with 4 µg female-derived C0t-1 DNA (2.0 uµg of each species). The FISH for CGH experiments followed the methodology described in [96]. Due to the lack of genomic DNA of the tataupa tinamou, CGH tests were not carried out in this species.

### Image analysis and microscopy

To corroborate 2n and FISH results, at least 30 metaphase spreads per individual were examined. Images were obtained with CoolSNAP on an Olympus BX50 microscope (Olympus Corporation, Ishikawa, Japan), and processed with Image-Pro Plus 4.1 software (Media Cybernetics, Silver Spring, MD, USA).

### Electronic supplementary material

Below is the link to the electronic supplementary material.

Supplementary Material 1

## Data Availability

The datasets generated during and/or analyzed during the current study are available in the NCBI database (https://www.ncbi.nlm.nih.gov/bioproject/) under accession numbers OR813804-OR813827. All data generated or analyzed during this study are included in this published article [and its supplementary information files].

## Abbreviations

Mya: Million years
BAC: Bacterial Artificial Chromosome
satDNA: Satellite DNA
TAREAN: Tandem Repeat Analyzer
RUL: Repeat unit lengths
DNO: *Dromaius novaehollandiae*
RAM: *Rhea americana*
CTA: *Crypturellus tataupa*
DnoSatDNA: *Dromaius novaehollandiae* satellite DNA
FISH: Fluorescence in situ Hybridization
DAPI: 4’,6-diamidino-2-phenylindole
gDNAs: Genomic DNA
CGH: Comparative genomic hybridization
PAR: Pseudoautosomal region
ICMBIO: Chico Mendes Institute of Biodiversity Conservation
ISBIO: Brazilian Biodiversity Information and Authorization System
DEMEM: Modified Eagle Medium
CEUA: Ethics Committee on Animal Experimentation
SRA: Sequence Read Archive
PCR: Polymerase Chain Reaction
