# Supplementary material for: Evolution of bird sex chromosomes: a cytogenomic approach in Palaeognathae species
Source: BMC Ecol Evol. 2024 Apr 23;24:51. doi: 10.1186/s12862-024-02230-5 (PMC11036779; doi:10.1186/s12862-024-02230-5)
Supplement: Supplementary file 1 — Supplementary Material 1 [file 12862_2024_2230_MOESM1_ESM.docx]

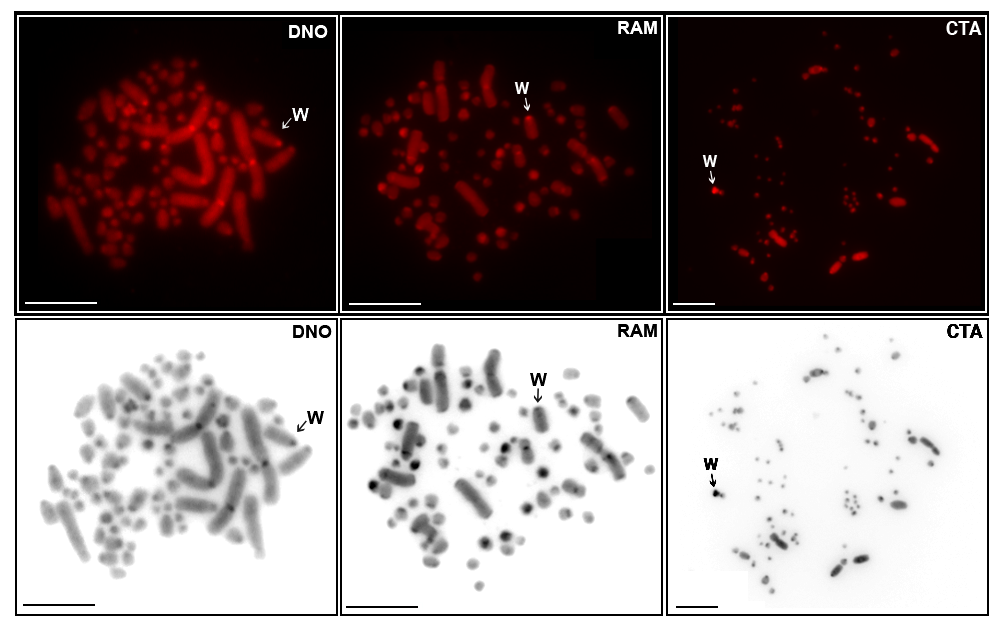


**Figure S1.** Female metaphase plates of the emu (DNO), the greater rhea (RAM), and the tatuapa tinamou (CTA) highlighting the distribution of C-positive heterochromatin. The upper figures depict the metaphases counterstained with propidium iodide while the lower ones were converted into gray-scale in addition to brightness/contrast adjustments. While the W chromosome was appropriately identified by a sequential hybridization with the microsatellite (GA)15, the Z chromosome could not be properly identified. Bar = 10 μm.


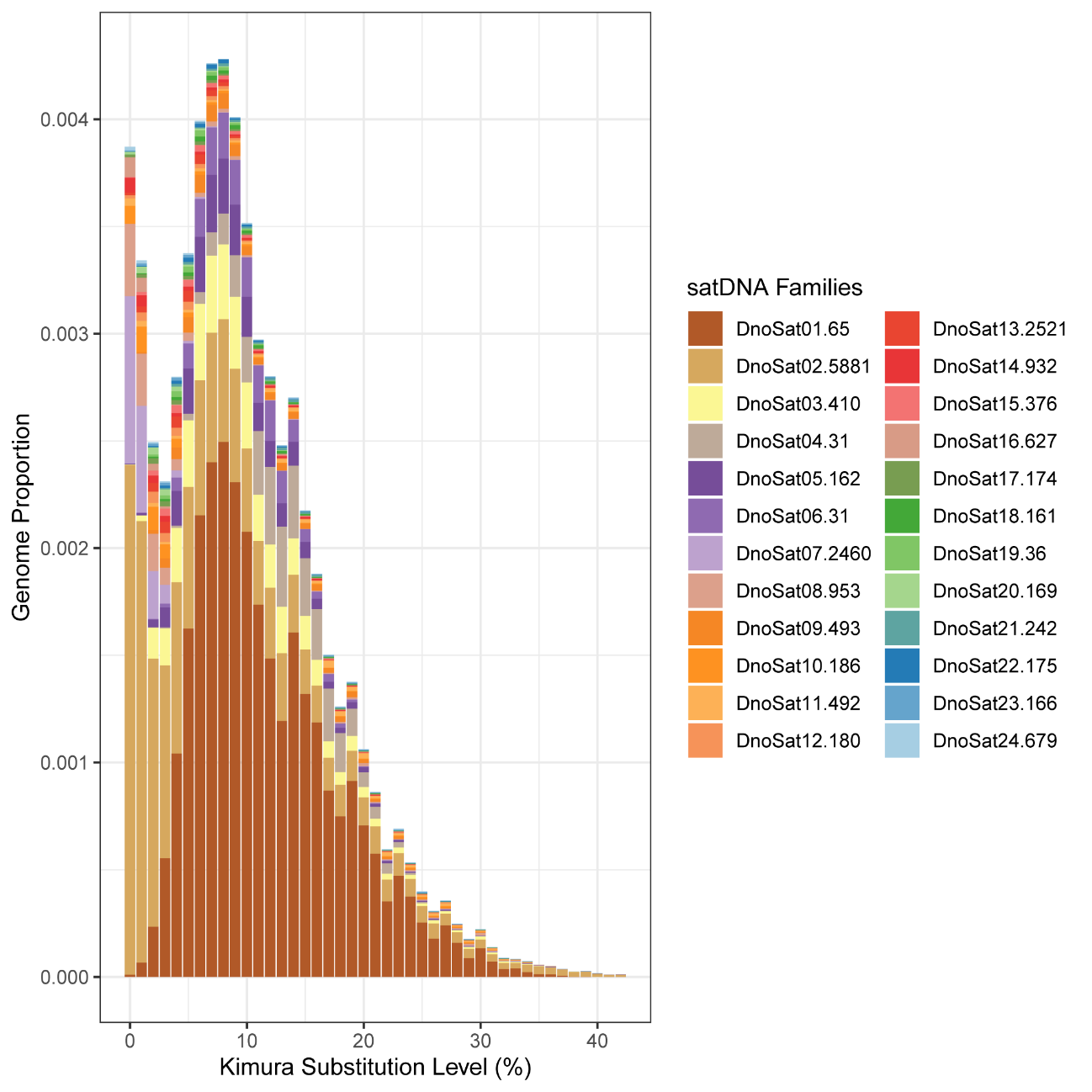


**Figure S2.** Repeat landscapes illustrating the distribution and divergence of all DnoSatDNA families.


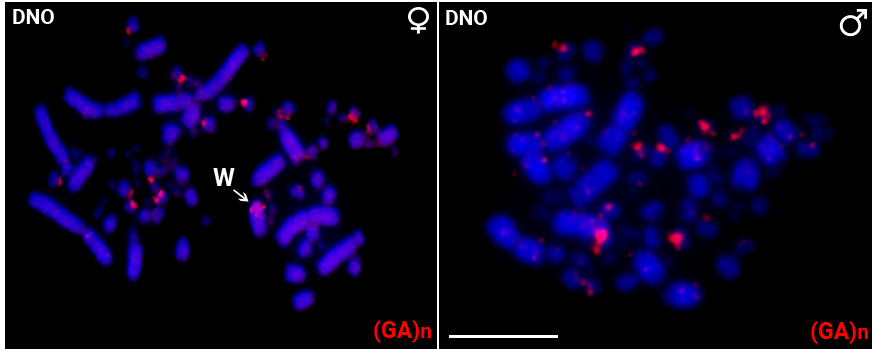


**Figure S3.** Female and male metaphase plates of the emu highlighting the chromosomal location of the microsatellite (GA)n. It should be noted that a single macrochromosome, found solely in females, exhibits a strong (GA)n signal in the pericentromeric region. Since there is no counterpart in males, we may therefore assume that it corresponds to the W chromosome (arrowed). Bar = 10 μm

**Sup Table 1:** Main characteristics of the emu satDNAs.

| **satDNA family** | **RUL** | **Abundance (M)** | **Abundance (F)** | **Abundance (F/M)** | **A+T (%)** |
| --- | --- | --- | --- | --- | --- |
| DnoSat01-65 | 65 | 0.024141958 | 0.029768812 | 1.233073613 | 36.90% |
| DnoSat02-5881 | 5881 | 0.014855083 | 0.013987972 | 0.941628675 | 56.60% |
| DnoSat03-410 | 410 | 0.002765063 | 0.004161444 | 1.50500839 | 41.50% |
| DnoSat04-31 | 31 | 0.003485776 | 0.003419289 | 0.980926163 | 42.00% |
| DnoSat05-162 | 162 | 0.003453255 | 0.002494376 | 0.722326013 | 40.70% |
| DnoSat06-31 | 31 | 0.002542566 | 0.001998162 | 0.785884068 | 35.50% |
| DnoSat07-2460 | 2460 | 0.000909707 | 0.001696275 | 1.864639248 | 52.00% |
| DnoSat08-953 | 953 | 0.000905984 | 0.001130665 | 1.247996818 | 39.90% |
| DnoSat09-493 | 493 | 0.000503838 | 0.00084015 | 1.667502496 | 40.80% |
| DnoSat10-186 | 186 | 0.00087018 | 0.000600285 | 0.689840047 | 39.20% |
| DnoSat11-492 | 492 | 0.00025661 | 0.000457174 | 1.781592431 | 41.10% |
| DnoSat12-180 | 180 | 0.000418681 | 0.000364115 | 0.869670914 | 37.80% |
| DnoSat13-2521 | 2521 | 0.00025421 | 0.000359398 | 1.413784508 | 41.30% |
| DnoSat14-932 | 932 | 0.000467166 | 0.000342343 | 0.732806599 | 28.60% |
| DnoSat15-376 | 376 | 0.000304624 | 0.000279255 | 0.916722462 | 42.30% |
| DnoSat16-627 | 627 | 0.000163236 | 0.000234889 | 1.438957226 | 39.70% |
| DnoSat17-174 | 174 | 0.000221117 | 0.00022 | 0.994949133 | 43.10% |
| DnoSat18-161 | 161 | 9.59525E-05 | 0.000207653 | 2.164128281 | 31.10% |
| DnoSat19-36 | 36 | 0.000386925 | 0.000170505 | 0.44066695 | 44.40% |
| DnoSat20-169 | 169 | 0.000542337 | 0.00016956 | 0.312647875 | 45.60% |
| DnoSat21-242 | 242 | 0.000153507 | 0.000166089 | 1.081964887 | 44.20% |
| DnoSat22-175 | 175 | 0.000216786 | 0.000150984 | 0.696465924 | 45.10% |
| DnoSat23-166 | 166 | 0.000121372 | 0.000143113 | 1.179123228 | 47.00% |
| DnoSat24-679 | 679 | 0.000144836 | 0.000142657 | 0.984960761 | 36.70% |
